# Impact of microbial biotransformation on *Zygophyllum decumbens* delile through comparative metabolic insights and evaluation of antihyperglycemic and antimicrobial activities

**DOI:** 10.1038/s41598-025-99590-9

**Published:** 2025-05-09

**Authors:** Alaadin E. El-Haddad, Soad M. Abd El-Khalik, Lina J. M. Abdel-Hafez, Jaky T. Zaki, Nagwan M. Gabr

**Affiliations:** 1 Pharmacognosy Department, Faculty of Pharmacy, October 6 University, Giza, 12585 Egypt; 2 Pharmacognosy Department, Faculty of Pharmacy, Helwan University, Cairo, 11795 Egypt; 3 Microbiology & Immunology Department, Faculty of Pharmacy, October 6 University, Giza, 12585 Egypt

**Keywords:** Metabolomics, Natural products, Medicinal chemistry, Pharmacology

## Abstract

*Zygophyllum* is known to be used traditionally in the management of several medical conditions, including diabetes, infections, and hypertension. This study aims to monitor the effect of microbial biotransformation on both antihyperglycemic and antimicrobial activities of *Zygophyllum decumbens* Delile using *Aspergillus niger.* The metabolic profile before and after biotransformation of the ethyl acetate extract was compared using HPLC-ESI-TOF-MS/MS. Pancreatic lipase, α-amylase, and α-glucosidase in vitro inhibition assays and agar diffusion method were used to evaluate the antihyperglycemic and antimicrobial activities, respectively. Eighty-six metabolites, mainly flavonoids and phenolic acids, with a few miscellaneous metabolites, were tentatively identified in both tested extracts. A 36% potentiation in antihyperglycemic activity was observed following microbial biotransformation. Regarding antimicrobial activity, the minimum inhibitory concentration (MIC) against both *E. coli* and *P. aeruginosa* decreased from 0.62 to 0.31 mg/ml, representing a nearly 50% reduction. It was noticed that microbial biotransformation altered 27 of the identified metabolites, which may contribute to the enhanced biological activities detected. The study depicts the first report of metabolic profiles and biological differences of *Z. decumbens* before and after biotransformation. It also validates the traditional use of *Z. decumbens* for hyperglycemia and its potential as a natural antimicrobial source.

**Supplementary Information:**

The online version contains supplementary material available at 10.1038/s41598-025-99590-9.

## Introduction

Diabetes mellitus is an endocrine metabolic disorder associated with abnormally elevated blood glucose levels. It is among the leading causes of death globally, as unmanaged diabetes can lead to ketoacidosis, which, if untreated, may result in stupor, coma, and ultimately death^1^. The American Diabetes Association has classified diabetes into three types: type 1, type 2, and gestational diabetes^2^. Patients with type 2 are recommended to receive alpha-glucosidase inhibitors as they need to slow down the release of glucose from polysaccharides thus delaying glucose absorption and causing a reduction in the blood glucose level^3,4^. Whereas patients with type 1 suffer from progressive destruction of the insulin-producing beta cells present in the pancreas, caused by lipid accumulation in the pancreas. Thus, lipase inhibitors are highly recommended to protect the pancreas by reducing lipid absorption, enabling the beta cells to produce normal insulin levels^5^.

Flavonoids, polyphenols, and organic acids are known to help alleviate oxidative stress-related conditions like obesity, liver damage, and diabetes, due to their antioxidant properties^6^, besides being described as glucosidase and amylase inhibitors in hyperglycaemia therapy^7,8^.

Beyond their metabolic benefits, phenolic compounds and organic acids have also demonstrated antimicrobial activity against various pathogenic microorganisms. This dual action has increased interest in medicinal plants for treating chronic conditions like diabetes and as promising natural alternatives to synthetic drugs in addressing antibiotic resistance^6,9^.

*Zygophyllum* is the genus in family Zygophyllaceae, containing around 80 species^10^. The extensive use of plants of the genus *Zygophyllum* in traditional medicine to manage diabetes, different fungal infections, hypertension, gout, and rheumatism is attributed to its bioactive compounds^11^. Reviewing the current literature revealed several reports concerning the beneficial phytochemicals, especially phenolic compounds, in different *Zygophyllum* species. *Z. album*,* Z. simplex*,* Z. dumosum*, and *Z. fabago* are reported to be rich in quercetin, kaempferol, and isorhamnetin, with their glycosides^10–16^.

Microbial biotransformation is an effective approach for generating more bioactive compounds through chemical transformation reactions that introduce chemo-, regio-, and/or stereochemical diversity into the molecular structure^17^. Microbial biotransformation of phenolic compounds has attracted attention due to its diverse enzyme systems and efficient production of high-value products. Such a process is considered to be non-polluting and friendly to the environment^18,19^. *Aspergillus niger* is a fungus that is widely used in the microbial biotransformation of various phytoconstituents, particularly flavonoids and phenolic acids^20^. Its mechanism may involve the generation of transformed products through processes such as hydroxylation, methylation, and dehydrogenation, facilitated by its enzymes produced^21–23^.

*Zygophyllum decumbens* is a subshrub native to the Egyptian deserts, known for its extensive traditional use^24^. *Z. decumbens* has been traditionally used in folk medicine for its anti-inflammatory, analgesic, and diuretic properties^25^. It is also used as a remedy for fever and gastrointestinal disturbances in traditional medicine systems^26^. However, limited research exists on its metabolic profile, and no studies have examined its biological activities^11,27,28^. No prior studies have investigated the microbial biotransformation of *Z. decumbens* or evaluated its chemical profile and biological activities before and after the process. HRLC-ESI-TOF-MS/MS technique was used to compare the chemical profiles of the studied extracts. The antihyperglycemic activity was evaluated using pancreatic lipase, alpha-amylase, and alpha-glucosidase inhibition assays. Moreover, the antimicrobial activity was evaluated using the agar diffusion method with MIC determination.

## Results

### Metabolites profiling

**Fig. 1:**
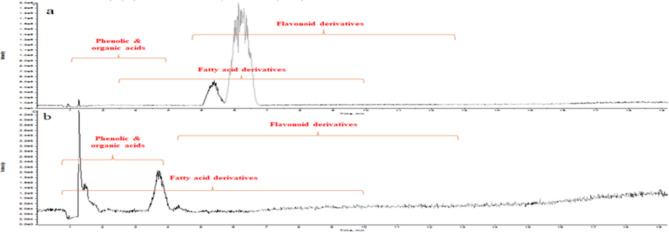
Total ion chromatogram of (**a**) ET extract, (**b**) ETM extract of the aerial parts of *Z. decumbens* in negative mode.

**Fig. 2:**
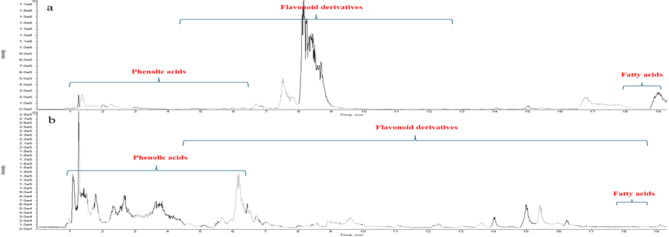
Total ion chromatogram of (**a**) ET extract, (**b**) ETM extract of the aerial parts of *Z. decumbens* in positive mode.

The metabolites of the ethyl acetate extracts of *Z. decumbens* aerial parts before (ET) and after (ETM) microbial transformation were investigated based on HRLC-ESI-TOF-MS/MS and their relevant databases. Detected compounds were tentatively identified by comparing their chromatographic behavior, retention time (R_t_), m/z values in total ion chromatogram (TIC) in both negative and positive modes, and their fragmentation patterns with those described in the literature review, MassBank, FoodB Database, and ReSpect database for plant extracts. The results in Table 1, Supplementary Table (1 S), Supplementary Figures (1 S), and Figs. 1 and 2, display eighty-six metabolites tentatively identified in both analyzed extracts. Fifty-nine of these metabolites correspond to ethyl acetate extract before microbial biotransformation (ET), forty-seven identified metabolites detected in ethyl acetate extract after microbial biotransformation (ETM) were identical to those detected in ET extract, in addition to twenty-seven metabolites that were newly emerged. Generally, the detected metabolites were dominated by flavonoids and phenolic acids, as shown in Supplementary Figures (2–14 S), along with a few organic acids, fatty acids, and other miscellaneous compounds.

**Table 1:** List of numbers of tentatively identified metabolites in ET & ETM extracts of *Z. decumbens*.

The class of compounds identified	The number of common compounds tentatively identified in both ET & ETM extracts	Total number of compounds tentatively identified in ET extract	Total number of compounds tentatively identified in ETM extract
Flavonoid derivatives	24	30	32
Phenolic acids	8	11	12
Organic acids	3	3	7
Fatty acids	4	5	11
Miscellaneous compounds	8	10	11

**Fig. 3:**
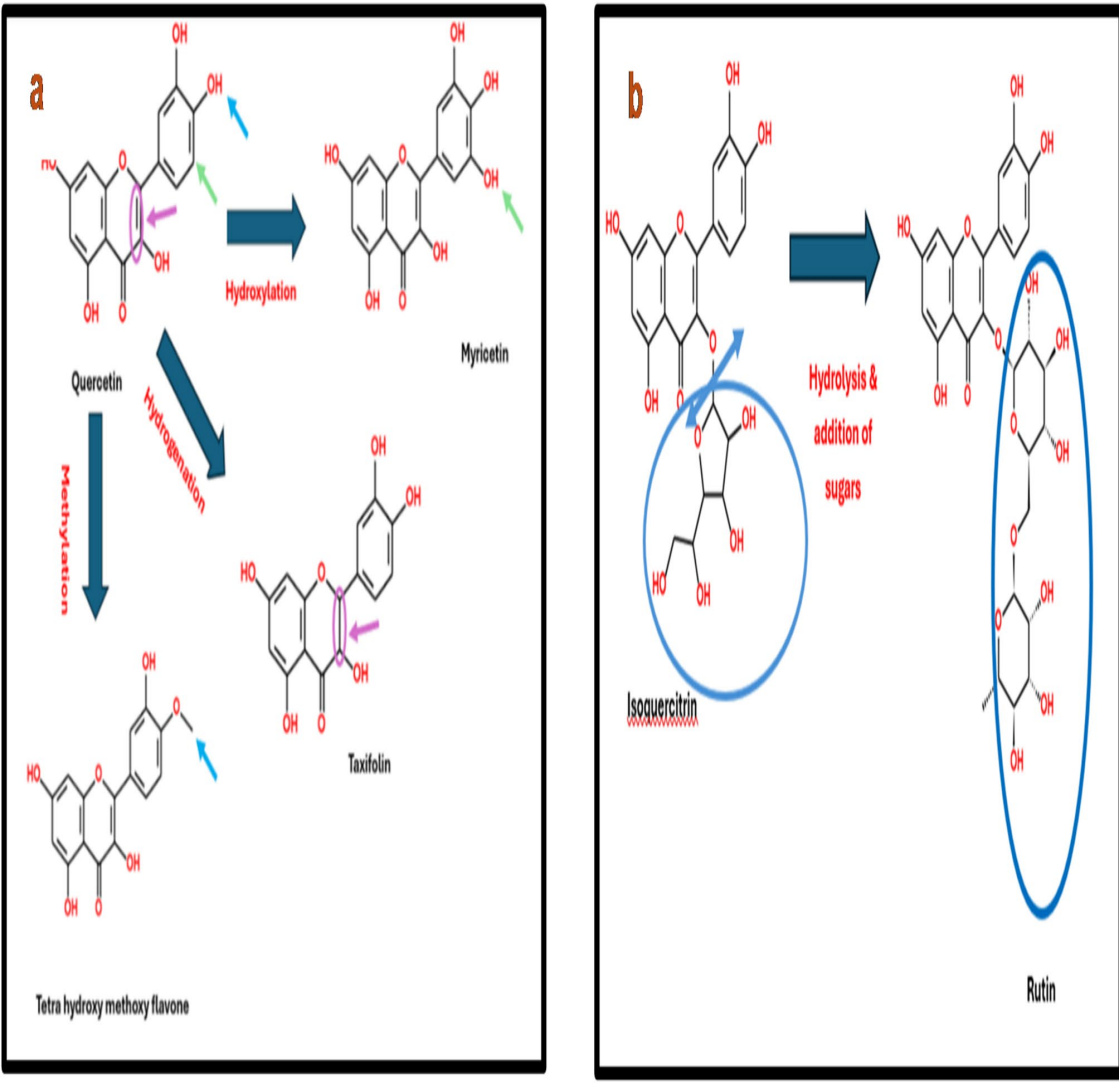
(**a** and **b**) Suggested biotransformation of quercetin and isoquercitrin by *A. niger*.

Thirty-eight of the tentatively identified compounds are flavonoid derivatives, as shown in Supplementary Table (1 S). Twenty-four out of the thirty-eight flavonoid derivatives were present in both ET and ETM extracts. However, six compounds, namely quercetin-*O*-di-hexoside (10), kaempferol-*O*-deoxyhexoyl-hexoside (12), luteolin-*O*-hexoside (19), quercitrin (23), dihydroxy methoxy flavone (35), and hyperoside (26), were detected exclusively in ET extract. In contrast, eight compounds, including kaempferol-*O*-deoxyhexoyl-hexoside-*O*-deoxyhexoside (1) Figure (2 S), rutin (5) Figure (3 S), acacetin-*O*-deoxyhexoyl-hexoside (7) Figure (4 S), kaempferol-*O*-pentoside (3) Figure (5 S), kaempferol-*O*-deoxyhexoside (8) Figure (6 S), taxifolin (6) Figure (7 S), myricetin (25) Figure (8 S), and tetrahydroxy methoxy flavone (38) Figure (9 S), appeared only in ETM extract.

Fifteen of the tentatively identified compounds are phenolic acid derivatives Supplementary Table (1 S). Eight of those phenolic acid derivatives are common in both ET and ETM extracts. However, sinapic (40), hydroxybenzoic (41), and salicylic acids (48) were detected only in ET extract, while hydroxy phenylacetic (52) Figure (10 S), quinic (45) Figure (11 S), benzoic (50) Figure (12 S), methoxy cinnamic acids (53) Figure (13 S), and cinnamate ester (55) Figure (14 S) emerged after microbial biotransformation in ETM extract.

### Determination of antihyperglycemic activity

The results in Table 2 showed that ET extract exhibits significant pancreatic lipase, α-glucosidase, and α-amylase inhibitory activities compared to reference standard drugs (orlistat and acarbose, respectively)^29^. The activity in all three enzyme assays increased in a dose-dependent manner. Following microbial biotransformation by *A. niger*, the ETM extract showed a 36% enhancement in α-glucosidase inhibitory activity at a dose of 100 µg/ml and a 9% enhancement at 1000 µg/ml. However, a reduction in α-amylase inhibitory activity was observed after biotransformation, alongside a non-significant change in pancreatic lipase inhibition.

**Table 2:** Antihyperglycemic activity of ET & ETM extracts via lipase inhibitory, alpha-amylase inhibitory, & alpha-glucosidase inhibitory assays:

Samples	% Inhibition					
	Lipase inhibitory assay		α-amylase inhibitory assay		α-glucosidase inhibitory assay	
	50µg/ml	500µg/ml	50 µg/ml	500 µg/ml	100 µg/ml	1000 µg/ml
ET	61.18 ± 6.78^a^	87.73 ± 6.19^*/ac^	19.67 ± 0.29^a^	49.71 ± 3.89^a^	46.79 ± 2.46^a^	63.89 ± 3.28^ac^
ETM	56.05 ± 1.52^c^	69.73 ± 3.65^a^	NA	19.05 ± 1.74^b^	63.74 ± 3.76^b^	69.68 ± 3.22^a^
Reference Standard drugs	Orlistat		Acarbose		Acarbose	
	5*µ*g/ml	50*µ*g/ml	0.1 *µ*g/ml	1 *µ*g/ml	15.625 *µ*g/ml	250 *µ*g/ml
	45.90 ± 2.10^c^	83.21 ± 0.41^c^	3.93 ± 0.69^c^	51.51 ± 4.30^a^	12.42 ± 1.42^c^	61.95 ± 1.54^c^

^*^ET extract was tested at 250 *µ*g/mL, representing 100% inhibition. Inhibition is expressed as mean ± standard deviation (SD), where *n* = 3 in three independent assays. NA: not active. Values of different letters are significantly different with *P* < 0.05.

### Determination of antimicrobial activity

Results presented in Table 3 show that only three microorganisms were susceptible to both ET^29^ and ETM extracts including two Gram-negative bacteria species (*E. coli* & *P. aeruginosa*) and one fungi species (*C. albican*) since a minimum inhibitory concentration (MIC) between 0.1 and 0.5 mg/ml is typically considered moderately active for crude plant extracts^30,31^. The zone of inhibition against *P. aeruginosa* of both tested extracts was nearly equal to that of Ciprofloxacin, used as a reference standard drug. The MIC for ETM extract obtained after microbial biotransformation was reduced to almost half that of ET extract against the two selected Gram-negative species (*E. coli* & *P. aeruginosa*). A 25% increment in the inhibition zone against *C. albicans* was observed for both tested extracts compared to Fluconazole as a control drug. However, MIC showed a 400% increment after microbial biotransformation, but still less than that of Fluconazole^32^.

**Table 3:** Antimicrobial activity of et & ETM extract of *Z. decumbens*.

Microorganism tested	ET		ETM		Fluconazole		Ciprofloxacin	
	Zone of inhibition (mm)	MIC (mg/ml)	Zone of inhibition (mm)	MIC (mg/ml)	Zone of inhibition (mm)	MIC (mg/ml)	Zone of inhibition (mm)	MIC (mg/ml)
*C. albicans*	25 ± 0.57^a^	0.156	25 ± 0.56^a^	0.625	20 ± 0.40^b^	0.747	–	–
*S. aureus*	–	–	–	–	–	–	30 ± 0.27	0.00976
*E. coli*	19 ± 0.60^a^	0.625	20 ± 0.50^a^	0.312	–	–	29 ± 0.35^b^	0.00976
*P. aeruginosa*	30 ± 0.56^a^	0.625	28 ± 0.45^b^	0.312	–	–	32 ± 0.25^c^	0.00976

Zone of inhibition expressed as mean ± SD (*n* = 3). Values of different letters are significantly different with *P* < 0.05.

## Discussion

The tentative identification of the flavonoid derivatives was based on the presence of the parent peak along with daughter peaks of the flavonoid aglycone in negative and/or positive ionization modes. The peak corresponding to the loss of one or more sugar moieties was also used for the identification. Compounds including eriodictyol-*O*- hexoside (13), isoquercitrin (17), gossypin (16), and naringenin- *O*- hexoside (24) were detected in the negative mode spectrum as deprotonated molecules at *m/z* 449.0987[M-H]^−^, 463.0862[M-H]^−^, 479.1079[M-H]^−^, 433.2072[M-H]^−^ respectively. While their daughter peaks corresponding to the neutral loss of 162 Da, representing the loss of [M-H-hexosyl]^−^ appeared *m/z* 287, 301, 317, and 271, respectively. Compounds like hesperidin (2), rhoifolin (9), kaempferol- *O* - deoxyhexosiyl – hexoside (12), and isorhamnetin - *O* - deoxyhexosyl – hexoside (14) showed a similar behavior in the negative ESI mode showing deprotonated molecules [M-H]^−^ at *m/z* 609.14, 577.12, 593.15, and 623.15 respectively. Their corresponding daughter ion peaks appeared at *m/z* 301, 269, 285 & 315 respectively, representing the loss of 308 Da, which corresponds to [M-H-deoxyhexoyl- hexoyl]^−^. In addition to the appearance of a daughter ion peak at *m/z* 301 [M-H-hexuronyl]^−^ due to neutral loss of 176 Da representing the hexuronyl moiety in compound (4)^33^.

Several other daughter peaks corresponding to the typical fragmentation patterns of flavonoids as reported in the literature review^33,34^ were detected in the spectrum of Luteolin-*O*-hexoside (19) at *m/z* 314 [Hexose + ^1,3^A^−^] and that of apigenin (34), which showed a characteristic peak appeared at *m/z* 93 due to loss of [ring B]^−^. Also, luteolin (28) showed a daughter peak appeared at *m/z* 161 corresponding to the loss of [^1,4^B^−^].

The remarkable ability of *A. niger* to efficiently utilize phenolic compounds may explain the emergence of the twelve compounds in the ETM extract, which are likely byproducts of the biotransformation process^35^. The nine phenolic compounds detected exclusively in ET extract are suggested to be consumed by *A. niger* which can utilize various mechanisms to bio-transform the chemical constituents within plant extracts^18,36^. This is evident in the biotransformation of quercetin (27)^37^ illustrated in Fig. 3a, and homogenestic acid (42)^38^ in the ET extract through the hydroxylation at position 5’ resulting in the formation of myricetin (25) and hydroxyphenyl acetic acid (52) respectively, both of which were identified in the ETM extract. Additionally, the emergence of tetrahydroxy methoxy flavone (38) and methoxy cinnamic acid (53) in the ETM extract after the methylation of one of the hydroxy groups in ring B at 3’ or 4’ of quercetin (27)^18^ as shown in Fig. 3a and *O*-methylation of coumaric acid (44)^35^ respectively. Furthermore, the hydrogenation of the double bond at position 2,3 in the parent nucleus of quercetin (27) was confirmed by the appearance of taxifolin (6)^18^, as shown in Fig. 3a.

Additional biotransformation mechanisms of *A. niger* were also observed in this study, including its ability to facilitate the addition and/or hydrolysis of sugar moieties^23,38^ which rationalizes the appearance of rutin (5) in the ETM extract from the biotransformation of isoquercitrin (17) as illustrated in Fig. 3b. As well as the appearance of kaempferol-*O*-deoxyhexosyl-hexoside-*O*-deoxyhexoside (1), and kaempferol-*O*-deoxyhexoside (8) in the ETM extract using kaempferol-*O*-deoxyhexosyl-hexoide (12) as a substrate undergoing addition of *O*-deoxyhexosyle and partial hydrolysis of the hexose moiety, respectively^23,38^.

The dehydroxylation ability of *A. niger* followed by the addition &/or hydrolysis of sugar moiety was clearly seen in the conversion of hesperidin (2), Kaempferol-*O*-hexuronide (22), and hydroxybenzoic acid (41) to their corresponding related derivatives acacetin-*O*-deoxyhexoyl-hexoside (7)^38^, Kaempferol-*O*-pentoside (3)^37^, and benzoic acid (50)^38^ respectively. It is worth mentioning that, while the transformation of a single compound by *A. niger* is well-studied for its ability to produce novel bioactive components, the transformation of complex mixtures has been seldom reported^39^.

The significant increase in the antihyperglycemic activity reflected by the improved α-glucosidase inhibitory activity of ETM extract may be attributed to the newly formed flavonoids and phenolic acids following microbial biotransformation, resulting in a synergistic effect. Both rutin and myricetin, two of the newly formed compounds, are reported to have individual significant α-glucosidase inhibitory activity^40,41^. Not only this, but the presence of quinic and methoxy cinnamic acids appearing after biotransformation is also reported to reduce the blood glucose level^42,43^. However, the marked decrease in α-amylase inhibitory activity can be attributed to the emerging organic acids detected after biotransformation, like maleic and hydroxybutyric acids, which chelate ions like Ca^2+,^ Mg²⁺, and Fe³⁺ through their carboxylic groups. Such ions are necessary cofactors for α-amylase inhibition^44,45^. These findings support the potential traditional use of *Z. decumbens* in the management of type 2 diabetes.

Moreover, the significant antimicrobial activity of the tested extracts against *E. coli*,* P. aeruginosa*,* and C. albicans* can be attributed to the presence of both phenolic acids and flavonoid derivatives. Those compounds are reported individually to have powerful antibacterial activity against Gram-negative strains. Quercetin and luteolin, detected in both extracts tested, are reported to be capable of increasing cytoplasmic membrane permeability, causing irregular cell shape, peptidoglycan, and cell membrane damage, in addition to decreasing nucleic acid content and increasing proteins inside the bacterial cells^46,47^. The emergence of benzoic and hydroxy phenylacetic acids in the ETM extract can justify the reduction in MIC to nearly half after biotransformation, as both compounds are reported individually as powerful antimicrobial agents against E. *coli* and *P. aeruginosa*^48^ compared to fluconazole as a reference standard drug. However, those phytoconstituents have no reported activity at all on the selected Gram-positive bacteria^47^, which rationalizes the resistance of *S. aureus* to ET and ETM extracts. Baicalein, luteolin, apigenin, myricetin, quercetin, quercitrin, isoquercitrin, and kaempferol, in addition to their corresponding glycosides, observed in both tested extracts, are reported to have individual powerful antifungal activity against different fungi, including *C. albicans*^49^. The combined presence of such compounds in both tested extracts produced a strong synergistic effect, significantly reducing the MIC compared to fluconazole, the reference standard^50^. The increase in MIC against *C. albicans* after microbial biotransformation may be explained by the detection of acacetin-*O*-deoxyhexosyl-hexoside in the ETM extract, which is reported to induce oxidative stress in fungal cells by generating reactive oxygen species (ROS). While elevated ROS levels can enhance antifungal activity, their presence may also interact with other flavonoids in the extract, potentially altering their structure and rendering them inactive, resulting in such an antagonizing effect^51^. Further studies with other microbes are recommended, aiming to discover novel drugs from natural products.

Those results provide useful data related to polyphenolic biotransformation, proving its great power in the conversion of active ingredients, and the enhancement of biological activities. The study depicts the first report of metabolic profiles and biological differences of *Z. decumbens* before and after biotransformation. It also validates the traditional use of *Z. decumbens* for hyperglycemia and its potential as a natural antimicrobial source.

## Materials and methods

### Plant material

*Zygophyllum decumbens* Delile aerial parts were collected from Wadi Hagul, Cairo-Suez Road, in April 2022 with the permission of the Agricultural Research Centre, Cairo, Egypt. It was collected under the supervision of and identified by Dr. A. Abd-Elmogali, a specialized taxonomist at Horticultural Research Institute, Flora & Phyto-taxonomy Research, Agricultural Research Centre, Dokki, Giza, Egypt. All methods involving plant materials were carried out per relevant institutional, national, and international guidelines and regulations. Necessary permissions for plant collection and experimental research were obtained from the Agricultural Research Centre, Giza, Egypt. The collected material matched the herbarium sheets deposited at Flora & Phyto-taxonomy Research Herbarium, Horticultural Research Institute, Agricultural Research Centre in Egypt, with a voucher number 4752 (CAIM).

### Chemicals and enzymes

All the chemicals used in this study were of analytical grade quality; Dimethyl sulfoxide (DMSO), potassium persulphate, methanol, methylene chloride, ethyl acetate, phosphate buffer, tris buffer, and standards (acarbose, orlistat, fluconazole & ciprofloxacin). The enzymes were purchased from Sigma-Aldrich; α-amylase from porcine pancreas (CAT number: A3176), α-glucosidase from *Saccharomyces cerevisiae* (CAT number: G5003) & pancreatic lipase from porcine pancreas (CAT number: L3126). The substrates were purchased from Sigma-Aldrich; 2-chloro-4-nitrophenyl-α-D-maltotrioside (CAT number: 93834), para nitrophenyl β-D-glucopyranoside (CAT number: N7006) & para 4-nitrophenyl dodecanoate (CAT number: 61716).

### Metabolite extraction

1 Kg of shade-dried aerial parts of *Z. decumbens* was extracted *via* cold maceration in aqueous methanol (7 × 3 L, 70%) at normal room temperature. The extract was then filtered using Whatman N◦1 filter paper, and the solvent was evaporated under reduced pressure using a rotary evaporator (Buchi, AG, Switzerland) at 40 °C, yielding 142 g of dry residues. Part of the extract (70 g) was suspended in distilled water (300 ml) and sequentially defatted with methylene chloride (200 ml × 3) followed by successive partitioning in ethyl acetate (200 ml × 3). Ethyl acetate extract was dried under reduced pressure using a rotary evaporator at 40 °C to obtain 1.7 g of dried residues named ET. The remaining 72 g of methanol extract was subjected to microbial biotransformation, followed by defatting and partitioning as described previously to obtain 0.92 g of dried residues named ETM.

### Microbial biotransformation

*Aspergillus niger ATCC 10,404* was collected from Microbiological Resources Centre (MIRCEN), Ain Shams University, Cairo, Egypt. The culture was subsequently grown in a conical flask with 100 ml Sabouraud Dextrose Broth (SDB) and incubated at 25 ± 2 °C for 14 days^52^. A spore suspension of *A. niger* was prepared according to established protocols and adjusted to 107 colony-forming spores per milliliter in PBS with the addition of Tween-20 (5 µl/ml) for liquid media inoculation^17^. The spore suspensions were incubated at 28 °C for 48 h on a rotary shaker at 180 rpm. A 5 ml portion of the older cultures was then subcultured into fresh SDB medium (30 ml). The methanol extract (70 g) was suspended in 20 ml of distilled water and subsequently added to 200 ml of SDB containing *A. niger* spores. The mixture was incubated for 14 days on a rotary incubator shaker at 180 rpm, with biotransformation progress monitored throughout the incubation period. Afterward, the culture was filtered, and the broth filtrate was extracted with ethyl acetate (3 × 1 L)^53^. A preliminary version of this experiment was conducted on a smaller scale prior to the large-scale trial, using 2 g of methanol extract suspended in 50 ml of SDB containing *A. niger* spores.

### HR LC-ESI-TOF-MS/MS analysis

The phytoconstituents of both ET and ETM extracts were analyzed by High-resolution liquid chromatography/ mass spectrometry (HR LC-ESI-TOF-MS/MS) as described by Abdel-Hamed et al., 2021^54^. Each of the tested extracts (50 mg) was individually dissolved in 1 ml of a solvent mixture containing water, methanol, and acetonitrile in a 50:25:25 (v/v) ratio. The extract solutions were sonicated for 10 min. & centrifuged (10,000 rpm, 10 min.). The extract solutions were sonicated for 10 min., followed by centrifugation at 10,000 rpm for an additional 10 min. Additionally, 50 µl of the stock solutions were diluted to 1000 µl using the reconstitution solvent mixture. Finally, 10 µl of each of the tested extracts was injected in both negative and positive modes. The results were compared to a blank sample. A 28-minute gradient elution program was employed for chromatographic separation at a flow rate of 0.3 ml/min. Two mobile phases, A & B, were used. Mobile phase A was composed of a 5 mM ammonium formate buffer, adjusted to pH 3.0 for positive mode and pH 8.0 for negative mode, in 1% methanol. Mobile phase B consisted of 100% acetonitrile for both positive and negative modes. HPLC separation was accomplished *via* ExionLC system (AB Sciex, Framing-ham, MA, USA) composed of; XSelect HSS T3 column (Waters Corporation, Milford, MA, USA) whose dimensions are 2.5 *µ*m, 2.1 × 150 mm, 0.5 *µ*m × 3.0 mm Phenomenex^®^ in-line filter disks & an autosampler system. The gradient elution was as follows: 100% A for 1 min, followed by a gradual increase from 100 to 10% A/B mixture in 20 min, then 10% A for 4 min, and ending with 10–100% A in 1.0 min, followed by re-equilibration with 100% A for 3 min. HPLC was integrated with a Triple TOF™ 5600 + system equipped with a Duo-Spray source operating in the electrospray ionization (ESI) mode for mass spectroscopy. In both positive and negative modes, the sprayer capillary and de-clustering potential voltages were set to ± 4500 V and ± 80 eV, respectively, at a temperature of 500 °C. The curtain gas was maintained at 25 psi, while gas 1 & gas 2 were set at 45 psi. Information-dependent acquisition (IDA) was utilized to obtain full-scan MS and MS/MS data. Its protocol was used to operate Triple TOF5600 + with ± 35 V collision energy for both positive & negative modes, 20 V CE spreading, and 10.000 ppm ion tolerance. Thus, representing high-resolution spectra across the mass with range between 50 and 1100 *m/z*. During the operation, the mass spectrometer represented a pattern where a 0.6502 s survey scan was detected, where the top 15 intense ions were selected for obtaining MS/MS fragmentation spectra after each scan.

### Data processing and annotation

MS-DIAL 4.9 (https://zenodo.org/records/12589462) was employed for data processing. Peakview software V1.2 (https://www.peakviewer.com) was used for the peak extraction from the total ion chromatogram (TIC). Tentative identification of the compounds in the tested extracts was based on comparing the compounds’ chromatographic behavior, retention time, m/z values, as well as the fragmentation patterns with those described in the literature review, MassBank (https://massbank.eu/MassBank/), Food Database (https://foodb.ca/), and ReSpect database (https://prime.psc.riken.jp/menta.cgi/prime/legacy_index) for phytochemicals.

### Determination of antihyperglycemic activity

#### Pancreatic lipase Inhibition assay

Lipase activity was assessed by measuring the conversion of *p*NPP into *p*-nitrophenol, which was monitored with a FluoStar Microplate Absorbance Reader (Omega, USA). Orlistat was used as a reference standard and tested at 10 and 100nM concentrations, whereas both tested extracts’ samples were initially dissolved in dimethyl sulfoxide (DMSO), and the final concentrations of 50 and 500 *µ*g/ml were prepared in methanol. The assay was carried out using a 96-microwell microplate (08- 772- 2 C, Fisher Scientific, USA). A 20 µl sample or blank was mixed with 140 µl of Tris buffer (pH 8). Subsequently, 20 µl of porcine lipase [Sigma-Aldrich, Germany] (2 mg/ml prepared in Tris buffer) was added, and the mixture was incubated at 37 °C for 10 min. Adding 20*µ*l *p*NPD solution (10 mM, in isopropanol) initiated the reaction. The mixture was then incubated for 30 min at 37ºC. Lipase activity was assessed by measuring the release of p-nitrophenol from its corresponding substrate at 405 nm using a FluoStar Omega microplate reader (USA)^55^.

#### α***-amylase Inhibition assay***

The stock solution of acarbose (reference standard drug used) was prepared at 0.1 and 1*µ*g/ml. ET and ETM extracts were tested at 50 and 500 *µ*g/ml as final concentrations in methanol. The assay was established in a 96-microwell microplate (08-772-2 C, Fisher Scientific, USA). 20 *µ*l of samples/blank were mixed with 140*µ*l phosphate buffer (50mM, 0.9% NaCl, pH7), followed by the addition of 20*µ*l amylase enzyme (1 mg/ml in phosphate buffer, pH7). The mixture was incubated for 15 min at 37 °C. 20 *µ*l substrate in phosphate buffer (0.375 mM) was then added. The mixture was incubated again for 10 min at 37 °C. Enzyme activity was determined by measuring the release of P-nitro-anilline at 405 nm using FluoStar Omega microplate reader (USA)^56^.

#### α**-glucosidase Inhibition assay**

Acarbose stock solution (2 Mm) in phosphate buffer (100 mM, pH 7) was prepared, and concentrations were prepared in water (15.625 and 250 *µ*g/ml) to obtain final concentrations (100 and 1000 *µ*g/ml) in methanol. The assay was carried out in a 96-microwell microplate (08- 772- 2 C, Fisher Scientific, USA). 25*µ*l samples /blank were incubated for 10 min at 37 °C with *α*-glucosidase obtained from *Saccharomyces cerevisiae* (50*µ*l, 0.6 U/mL) in phosphate buffer (0.1 M, pH 7). 25*µ*l pNPG (3 mM) was together with a substrate in phosphate buffer (pH 7) was then added, followed by further incubation of the mixture for 5 min at 37 °C. Glucosidase activity was determined by measuring the release of p-nitrophenol at 405 nm using FluoStar Omega microplate reader (USA)^55^. The percentage of inhibition of *α*-glucosidase, pancreatic lipase & *α*-amylase was calculated according to the equation:

$$\% {\text{ Inhibition}}=\left[ {\left( {{{\text{A}}_{{\text{blank}}}}--{{\text{A}}_{{\text{sample}}}}} \right)/{{\text{A}}_{{\text{blank}}}}} \right] \times {\text{ 1}}00$$

where: A_blank_ is the absorbance of the control (blank, without inhibitor), and A_sample_ is the absorbance in the presence of the inhibitor.

### Determination of antimicrobial activity

#### Materials

*Staphylococcus aureus ATCC 25,923*, *Pseudomonas aeruginosa ATCC 9027*, *Escherichia coli ATCC 8739*, and *Candida albicans ATCC 10231*were collected from the Microbiology Lab of the Faculty of Pharmacy, Cairo University. *S. aureus* was grown on Mueller Hinton agar (MHA) (DifcoTM, Strasbourg, France), and on Tryptic soya agar (TSA) (DifcoTM, Strasbourg, France) for *P. aeruginosa*, and *E. coli* for a duration of 24 h at 37 °C. *C. albicans* was grown on Sabouraud Dextrose Agar (SDA) (HiMedia Laboratories, 507 School House Rd., USA) for 48 h. at 25 °C. A single colony of each isolate was inoculated individually into 2 ml Mueller Hinton broth (MHB), Tryptic soya broth (TSB) & Sabouraud broth with an overnight incubation at 37 °C & 25 °C as mentioned above.

#### Cup agar diffusion method

The cup agar diffusion method was applied for the antimicrobial susceptibility testing^57^. All the microbial cultures were standardized to an optical density (OD) of 0.5 at 600 nm. MHA, TSA, and SDA plates were inoculated with 100 µl of the respective microbial isolates respectively. Additionally, 5 mm wells were filled with 100 µl of the tested extracts, dissolved in sterile water at a concentration of 5 mg/ml. The plates were then incubated for 24 h. at 37 °C for bacteria isolates & 48 h. at 25 °C for fungi isolates. The zone of inhibition was measured in mm. The standard deviation was calculated by determining the mean value of the zone of inhibition, obtained from repeating each test three times.

#### Broth micro-dilution method

The broth micro-dilution method is applied to measure antimicrobial susceptibility. The procedures involve the preparation of two-fold serial dilutions of the ET and ETM extracts with different concentrations (2.5-0.078 mg/ml) in a Mueller Hinton broth using a 96-well microtitration plate. Each well was then inoculated with a microbial inoculum of *S. aureus*,* E. coli*,* P. aeruginosa & C. albicans* prepared in the same medium after dilution of a standardized microbial suspension adjusted to 0.5 McFarland scale. Afterward, the 96-well microtitration plates were incubated under suitable conditions, following the test microorganism used as mentioned before. The minimum inhibitory concentration (MIC), which is defined as the lowest concentration of plant extracts that completely inhibits the growth of the organism in microdilution wells^58^, was then recorded for each microorganism.

### Statistical analysis

Data was expressed as the mean ± SEM for all the experiments, and statistical analysis was carried out by one-way ANOVA followed by the Tukey multiple comparisons test to calculate the significance of the difference between treatments. Values of *p* < 0.05 were considered significant. All statistical analyses were performed, and graphs were sketched using GraphPad Prism (ISI, USA) software V9 (https://www.graphpad.com/features/prism-anova). The values of different letters differ significantly.

## Electronic supplementary material

Below is the link to the electronic supplementary material.

Supplementary Material 1

## Data Availability

The datasets supporting the conclusions of this article are included within the article and the supplementary file.
