# Supplementary material for: Impact of microbial biotransformation on Zygophyllum decumbens delile through comparative metabolic insights and evaluation of antihyperglycemic and antimicrobial activities
Source: Sci Rep. 2025 May 9;15:16244. doi: 10.1038/s41598-025-99590-9 (PMC12064765; doi:10.1038/s41598-025-99590-9)
Supplement: Supplementary file 1 — Supplementary Material 1 [file 41598_2025_99590_MOESM1_ESM.pdf]

Supplementary file

Table (1S): Tentatively identified metabolites in ET & ETM extracts of *Z. decumbens* using LC-ESI-TOF-MS/MS (Negative/ Positive Modes):

No.	Rt (min.)	Identified metabolite	Exact Formula	[M-H]⁻	[M+H]⁺	Ms/Ms	Compounds detected in ET	Compounds detected in ETM
**Flavonoid derivatives**								
1	4.23	kaempferol-*O*-deoxyhexoyl-hexoside-*O*-deoxyhexoside	$C_{33}H_{40}O_{19}$	739.0338		285		√
2	4.43	Hesperidin	$C_{28}H_{34}O_{15}$	609.1427	611.1586	563, 301	√	√
3	4.48	Kaempferol-*O*-pentoside	$C_{20}H_{18}O_{10}$	417.1555		399, 285		√
4	4.64	Quercetin -*O*- hexuronide	$C_{21}H_{18}O_{13}$	477.1536		393, 301, 179, 273, 151	√	√
5	4.73	Rutin	$C_{27}H_{30}O_{16}$	609.1418		301		√
6	4.77	Taxifolin	$C_{15}H_{12}O_{7}$	303.1809		285, 257, 195		√
7	4.85	Acacetin-*O*-deoxyhexosyl-hexoside	$C_{28}H_{32}O_{14}$	591.9002		283, 445		√
8	5.33	Kaempferol-*O*- deoxyhexoside	$C_{21}H_{20}O_{10}$	431.1454		385, 285		√
9	5.53	Rhoifolin	$C_{27}H_{30}O_{14}$	577.1207		531, 269	√	√
10	5.54	Quercetin-*O*-di-hexoside	$C_{27}H_{30}O_{17}$	625.143		607, 301	√	
11	5.56	Baicalein-*O*- hexuronide	$C_{21}H_{18}O_{11}$	445.1728		399, 269, 205, 195	√	√
12	5.64	Kaempferol-*O*- deoxyhexosyl-hexoside	$C_{27}H_{30}O_{15}$	593.1508		285	√	
13	5.84	Eriodictyol-*O*- hexoside	$C_{21}H_{22}O_{11}$	449.0987	451.1963	403, 287, 179	√	√
14	5.91	Isorhamnetin-*O*- deoxyhexosyl-hexoside	$C_{28}H_{32}O_{16}$	623.1595	625.1817	315, 300	√	√
15	5.95	Isorhamnetin-*O*-hexoside	$C_{22}H_{22}O_{12}$	477.1029	479.1166	315, 300, 271	√	√
16	6.01	Gossypin	$C_{21}H_{20}O_{13}$	479.1079		317, 271, 243	√	√
17	6.02	Isoquercitrin	$C_{21}H_{20}O_{12}$	463.0862	465.2480	417, 301, 287, 283	√	√
18	6.71	Phlorizin	$C_{21}H_{24}O_{10}$	435.2259	437.1428	389, 273	√	√
19	6.72	Luteolin-*O*- hexoside	$C_{21}H_{20}O_{11}$	447.0929		401, 314, 285	√	
20	6.88	Syringetin-*O*- hexoside	$C_{23}H_{24}O_{13}$	507.1491		463, 355, 345, 327, 303, 85	√	√
21	7.15	Quercetin-*O*-pentoside	$C_{20}H_{18}O_{11}$	433.2085		387, 301	√	√
22	7.21	Kaempferol-*O*-hexuronide	$C_{21}H_{18}O_{12}$	461.1099		415, 285	√	√
23	7.54	Quercitrin	$C_{21}H_{20}O_{11}$	447.1337		401, 301	√	
24	8.31	Naringenin-*O*- hexoside	$C_{21}H_{22}O_{10}$	433.2072		271, 151	√	√
25	8.58	Myricetin	$C_{15}H_{10}O_{8}$	317.1109		209, 181		√
26	8.64	Hyperoside	$C_{21}H_{20}O_{12}$		465.2805	301	√	

Continuing: Table (1S): Tentatively identified metabolites in ET & ETM extracts of *Z. decumbens* using LC-ESI-TOF-MS/MS (Negative/ Positive Modes):

No.	Rt (min.)	Identified metabolite	Exact Formula	$[M-H]^-$	$[M+H]^+$	Ms/Ms	Compounds detected in ET	Compounds detected in ETM
27	8.69	Quercetin	$C_{15}H_{10}O_7$	301.1175	303.0505	283, 273, 259, 255, 229, 227, 193, 151	√	√
28	9.33	Luteolin	$C_{15}H_{10}O_6$	285.1237	287.0553	267, 243, 239, 229, 176, 161	√	√
29	9.37	Isorhamnetin	$C_{16}H_{12}O_7$	315.0549	317.0659	300, 271, 164	√	√
30	9.44	Naringenin	$C_{15}H_{12}O_5$	271.1072		253, 243, 197	√	√
31	9.69	Hesperetin	$C_{16}H_{14}O_6$	301.1172		283, 255, 193, 179, 151	√	√
32	10.10	Tetrahydroxy flavanone	$C_{15}H_{12}O_6$	286.9382	289.1335	259, 241, 180	√	√
33	10.36	Acacetin	$C_{16}H_{12}O_5$	283.1068	285.1133	268, 265, 237	√	√
34	11.07	Apigenin	$C_{15}H_{10}O_5$	269.2096		223, 93	√	√
35	11.68	Dihydroxy methoxy flavone	$C_{16}H_{14}O_5$		287.0547	269, 193	√	
36	12.68	Pentahydroxy flavan	$C_{15}H_{14}O_6$	288.955		181	√	√
37	12.90	Kaempferide	$C_{16}H_{12}O_6$	299.2022	301.1413	281, 253, 193	√	√
38	16.59	Tetrahydroxy methoxy flavone	$C_{16}H_{12}O_7$		317.1749	299, 150, 123, 109		√
				Phenolic acids				
No.	Rt (min.)	Identified metabolite	Exact Formula	$[M-H]^-$	$[M+H]^+$	Ms/Ms	Compounds detected in ET	Compounds detected in ETM
39	0.95	Gentisic acid	$C_7H_6O_4$	153.0209		109	√	√
40	1.02	Sinapic acid	$C_{11}H_{12}O_5$	223.0648	225.0849	205, 179, 163, 91	√	
41	1.06	Hydroxybenzoic acid	$C_7H_6O_3$	137.0246		93	√	
42	1.07	Homogentisic acid	$C_8H_8O_4$	167.0357		123, 108	√	√
43	1.10	Vanillic acid	$C_8H_8O_4$	167.0332		108, 123, 152	√	√
44	1.26	Coumaric acid	$C_9H_8O_3$	163.0407	165.0538	119, 93	√	√
45	1.27	Quinic acid	$C_7H_{12}O_6$	191.0675		173, 101		√
46	1.31	Ferulic acid	$C_{10}H_{10}O_4$	193.0493	195.0647	162, 149	√	√
47	1.42	Caffeic acid	$C_9H_8O_4$	179.0034		135, 71	√	√
48	1.52	Salicylic acid	$C_7H_6O_3$	137.0377		119, 93	√	
49	1.93	Sinapoyl malate	$C_{15}H_{16}O_9$	339.0857		321, 303, 295, 279, 207	√	√
50	1.98	Benzoic acid	$C_7H_6O_2$		123.0544	105, 78		√
51	2.18	Shikimic acid	$C_7H_{10}O_5$	173.0073		155, 131	√	√

Continuing: Table (1S): Tentatively identified metabolites in ET & ETM extracts of *Z. decumbens* using LC-ESI-TOF-MS/MS (Negative/ Positive Modes):

No.	Rt (min.)	Identified metabolite	Exact Formula	$[M-H]^-$	$[M+H]^+$	Ms/Ms	Compounds detected in ET	Compounds detected in ETM
52	2.64	Hydroxy phenylacetic acid	$C_8H_8O_3$	151.0370		107, 106, 93		√
53	3.92	Methoxy cinnamic acid	$C_{10}H_{10}O_3$		179.0779	161, 134, 107		√
				Fatty acids				
No.	Rt (min.)	Identified metabolite	Exact Formula	$[M-H]^-$	$[M+H]^+$	Ms/Ms	Compounds detected in ET	Compounds detected in ETM
54	4.10	Chlorogenic acid	$C_{16}H_{18}O_9$	353.0921		179, 175	√	√
55	5.81	Cinnamate	$C_9H_8O_2$	146.9949	149.0313	103		√
56	6.43	Sinapoyl hexoside	$C_{17}H_{22}O_{10}$		387.1639	225, 207	√	√
57	0.80	Methyl glutaric acid	$C_6H_{10}O_4$	145.0493		127, 101		√
58	0.81	Hydroxy methyl pentanoate	$C_6H_{12}O_3$	131.0337		113, 87		√
59	1.05	Citraconic acid	$C_5H_6O_4$	129.0006		85		√
60	1.18	Aconitate	$C_6H_6O_6$	173.0809		155, 137, 129, 83		√
61	1.56	Isopropyl malic acid	$C_7H_{12}O_5$	175.0702		157, 139, 85		√
62	2.69	Arachidonic acid	$C_{20}H_{32}O_2$	303.1335		258	√	
63	5.22	Sebacate	$C_{10}H_{18}O_4$	201.1128		183, 157, 156, 113		√
64	9.78	Linolenic acid	$C_{18}H_{30}O_2$	277.1442		233	√	√
65	18.42	Linoleic acid	$C_{18}H_{32}O_2$		281.1939	263	√	√
				Organic acids derivatives				
No.	Rt (min.)	Identified metabolite	Exact Formula	$[M-H]^-$	$[M+H]^+$	Ms/Ms	Compounds detected in ET	Compounds detected in ETM
66	0.82	Succinic acid	$C_4H_6O_4$	117.0192		99, 73	√	√
67	0.90	Lactic acid	$C_3H_6O_3$	89.026		71		√
68	0.95	Methyl lactic acid	$C_4H_8O_3$	103.0399		73, 59, 57	√	√
69	0.96	Maleic acid	$C_4H_4O_4$	115.0399		97, 71		√
70	0.98	Methylmalonic acid	$C_4H_6O_4$	117.0572		99, 73		√
71	1.01	Hydroxybutyric acid	$C_4H_8O_3$	103.0398		73, 59		√
72	1.015	Mucate	$C_6H_{10}O_8$	209.079		191, 165, 137, 121		√
73	2.90	Phenyl lactic acid	$C_9H_{10}O_3$	165.0211		147, 121	√	√
				Miscellaneous metabolites				
No.	Rt. (min.)	Identified metabolite	Exact Formula	$[M-H]^-$	$[M+H]^+$	Ms/Ms	Compounds detected in ET	Compounds detected in ETM
74	1.23	Citramalate	$C_5H_8O_5$	147.0298		129, 59, 57	√	√
75	1.23	Ketoisoleucine	$C_6H_{10}O_3$	129.0282		85, 69, 55	√	√

Continuing: Table (1S): Tentatively identified metabolites in ET & ETM extracts of *Z. decumbens* using LC-ESI-TOF-MS/MS (Negative/ Positive Modes):

No.	Rt (min.)	Identified metabolite	Exact Formula	$[M-H]^-$	$[M+H]^+$	Ms/Ms	Compounds detected in ET	Compounds detected in ETM
76	1.27	Hydroxy methyl glutaric acid	$C_6H_{10}O_5$	161.0831		143, 117, 99	√	√
77	1.72	Syringaldehyde	$C_9H_{10}O_4$		183.0946	165, 153, 123	√	√
78	2.18	Suberic acid	$C_8H_{14}O_4$	173.0073		155, 129, 85	√	√
79	2.31	Dihydroxy mandelate	$C_8H_8O_5$	183.0482		138	√	√
80	2.68	Hydroxy methoxy phenylacetic acid	$C_9H_{10}O_4$	181.0531		163, 151, 137		√
81	4.25	Hydroxy methoxy cinnamaldehyde	$C_{10}H_{10}O_3$		179.0827	161, 135, 124		√
82	5.49	Hydroxy methoxy mandelate	$C_9H_{10}O_5$	197.096		167, 153, 122		√
83	5.72	Esculin	$C_{15}H_{16}O_9$	339.1081		311, 293, 177	√	√
84	7.02	Sinapyl aldehyde	$C_{11}H_{12}O_4$	207.071		189, 178	√	√
85	7.26	Dihydroxy coumarin	$C_9H_6O_4$	177.0262		133	√	
86	8.30	Cinnamaldehyde	C_9H_8O		133.101	103, 77	√	

All fragmentation was firstly applied according to ESI negative mode if available, the error in metabolites identification was within ± 10 ppm & highlighted compounds represent changed compounds appearing after microbial biotransformation (ETM)

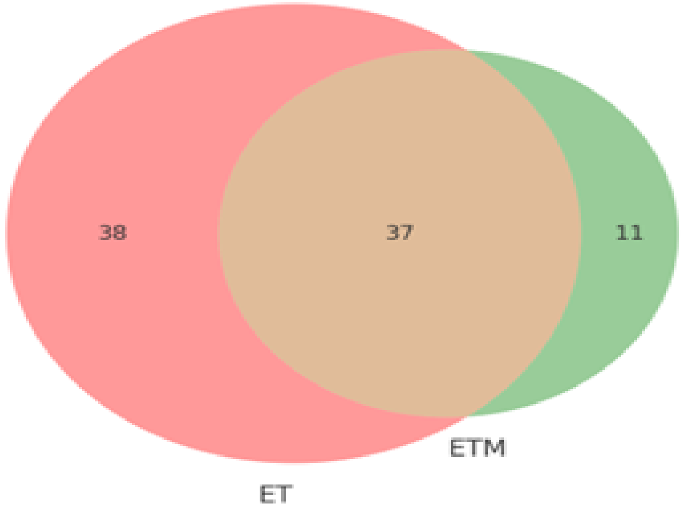

Figure (1S): Venn Diagram of the number of compounds detected in ET & ETM extracts of *Z. decumbens*

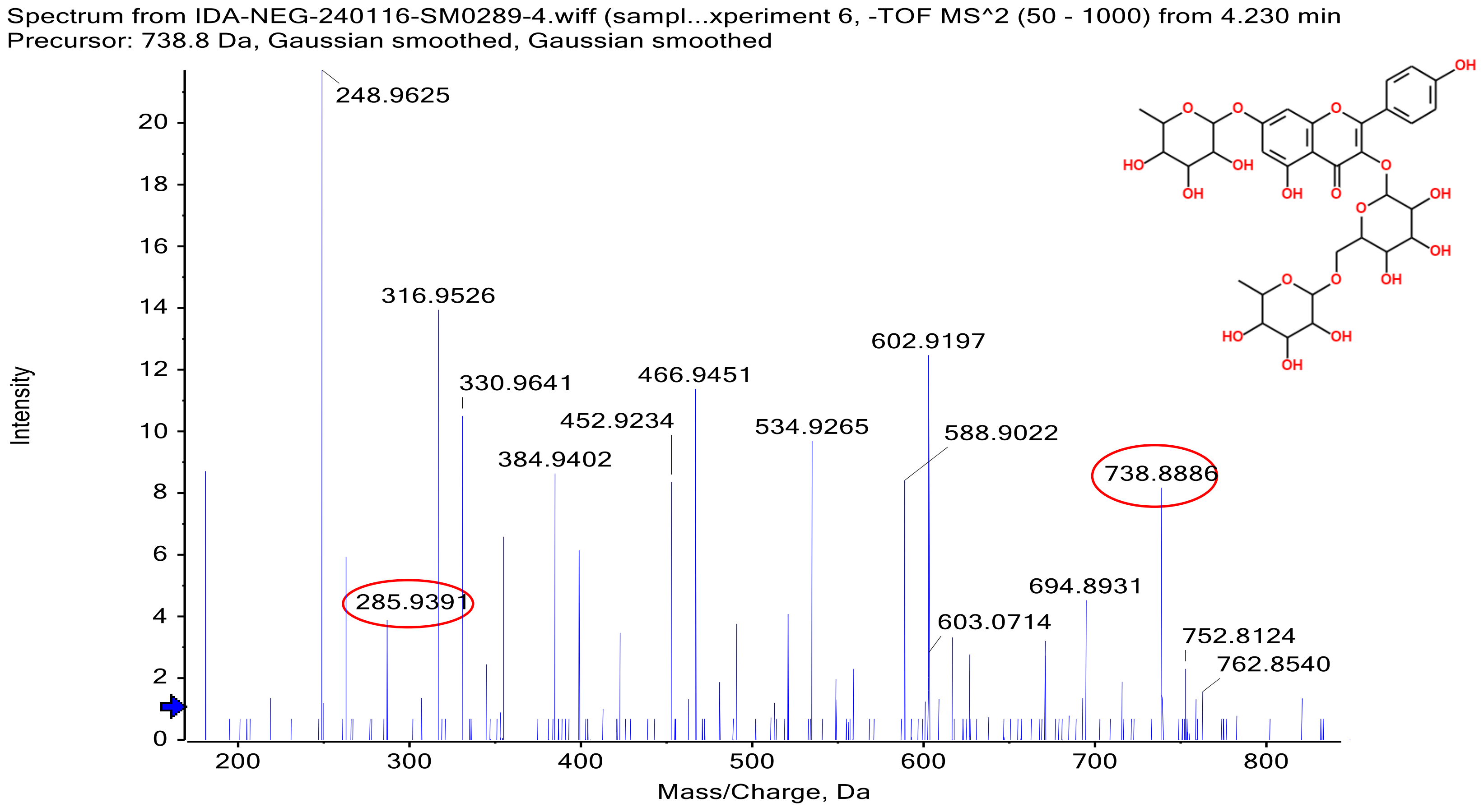

Figure (2S): LC/Ms-Ms of kaempferol-*O*-deoxyhexoyl-hexosyl-*O*-deoxyhexoside of ETM in ESI negative mode

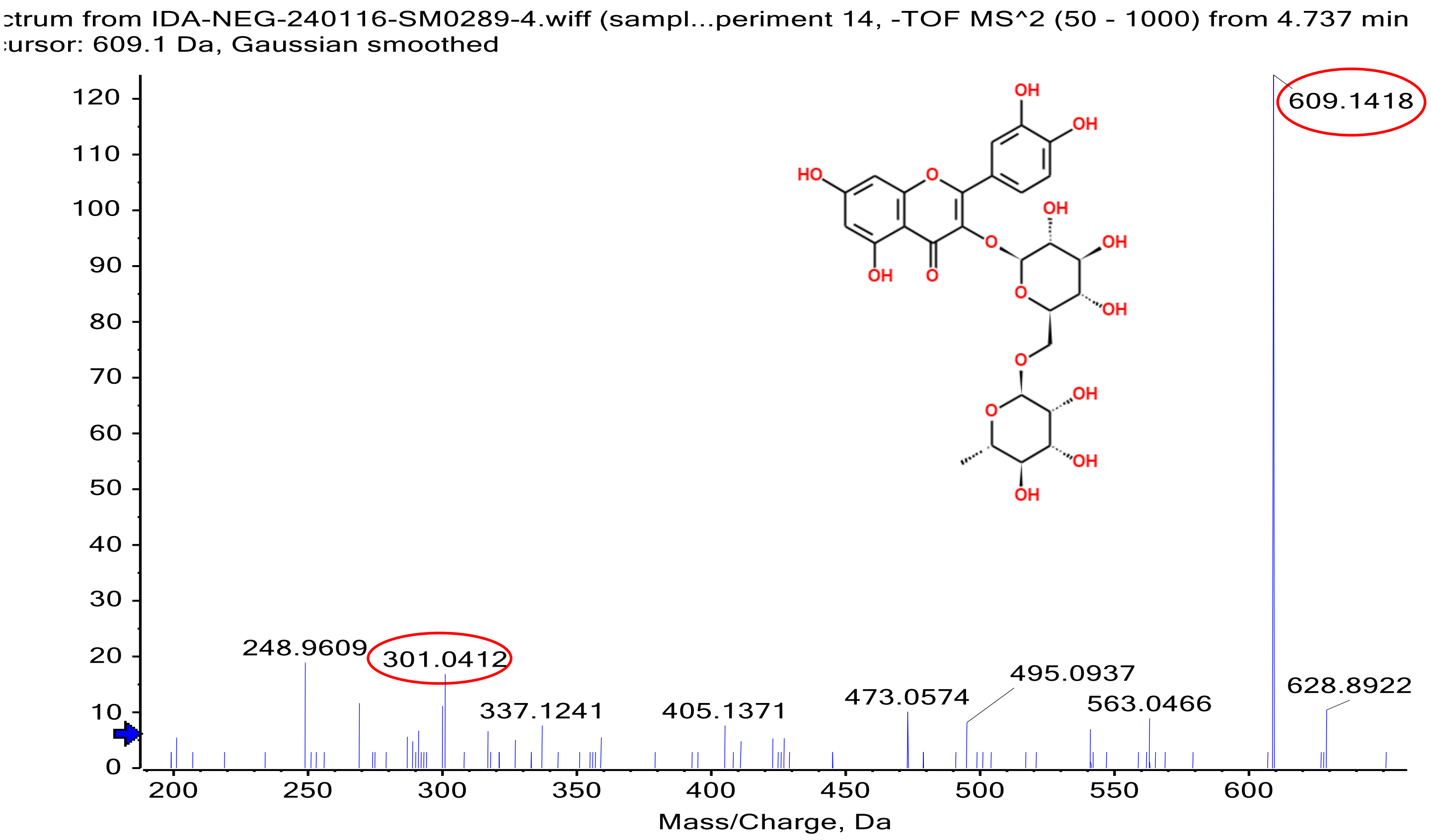

Figure (3S): LC/Ms-Ms of rutin of ETM in ESI negative mode

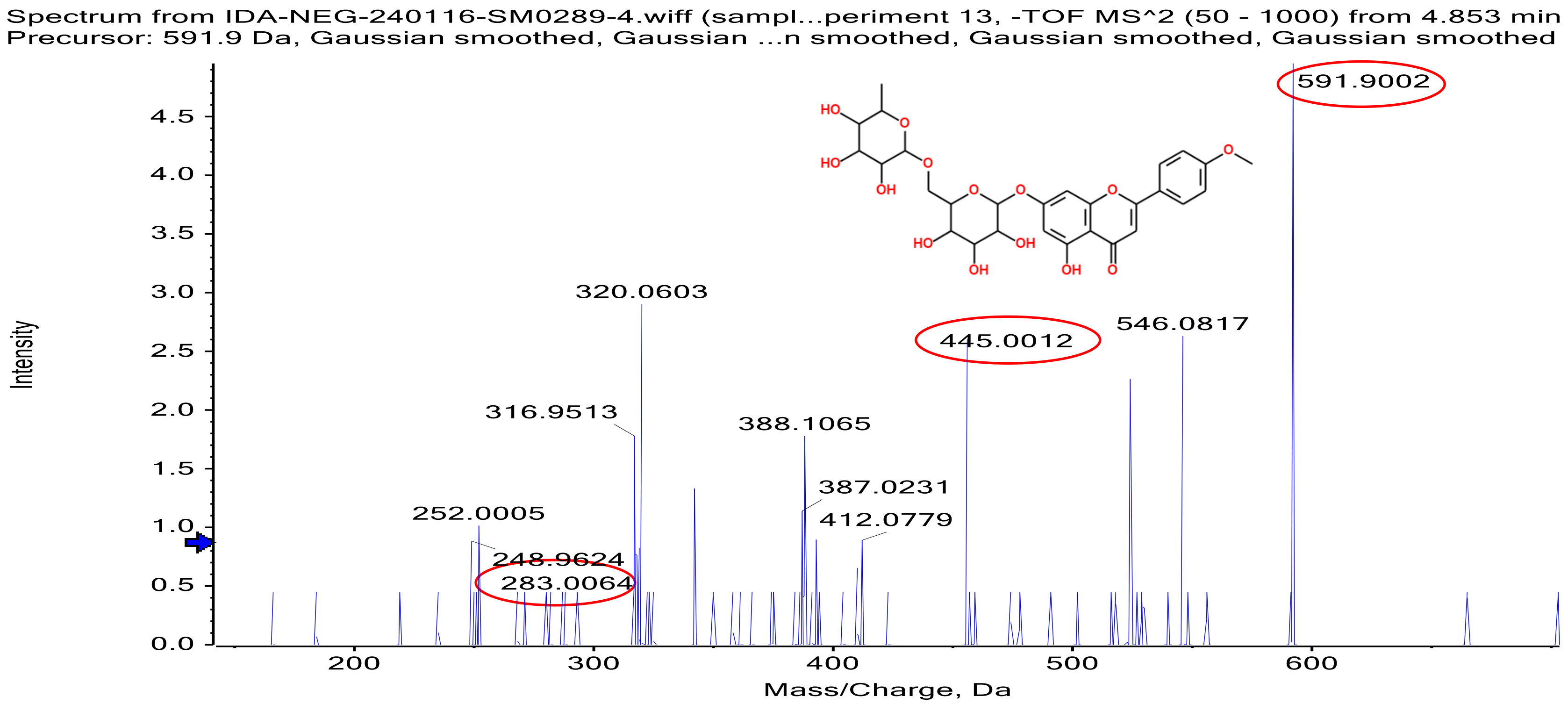

Figure (4S): LC/Ms-Ms of Acacetin-*O*-deoxyhexosyl-hexoside of ETM in ESI negative mode

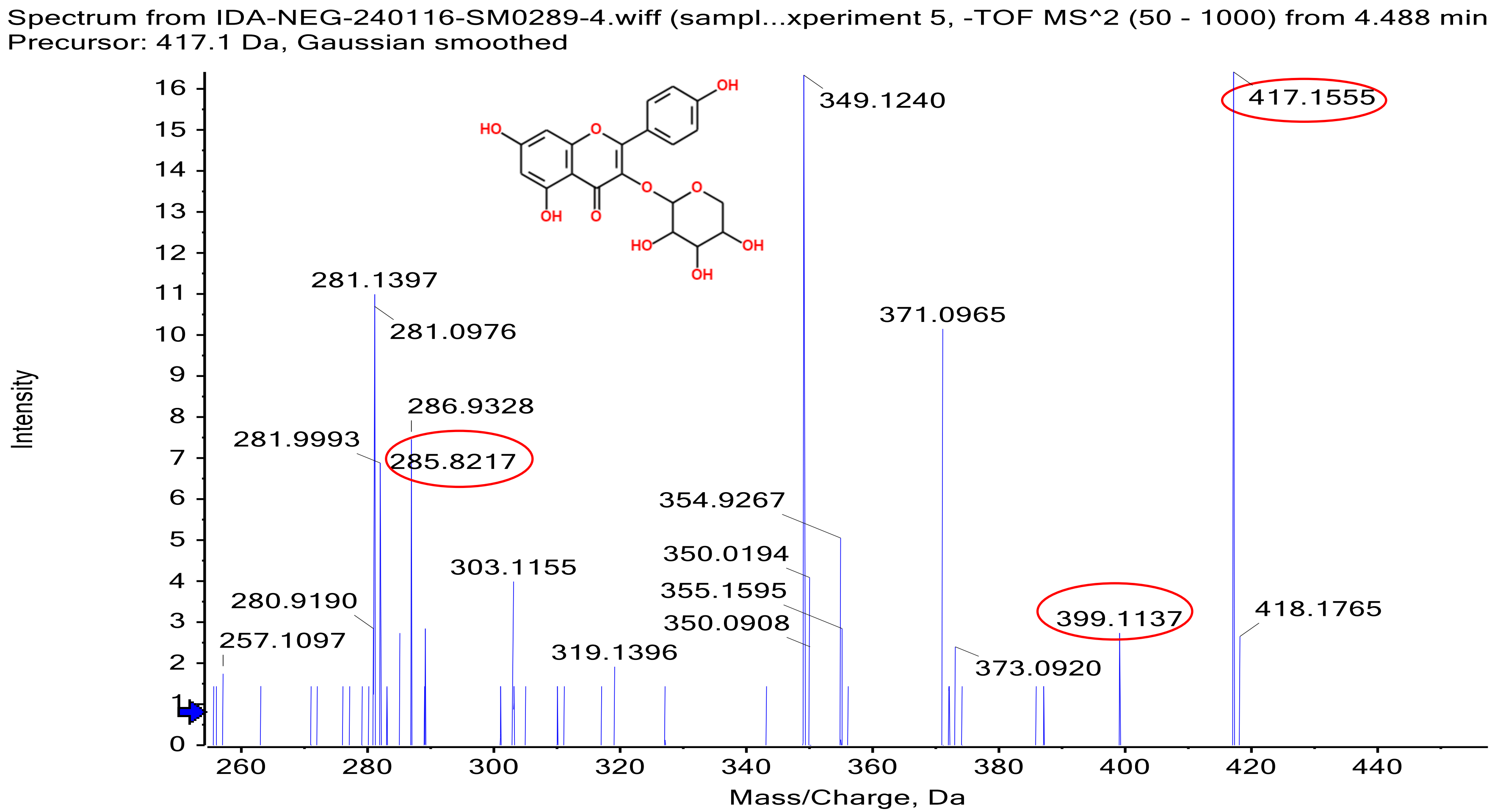

Figure (5S): LC/Ms-Ms of kaempferol-*O*-pentoside of ETM in ESI negative mode

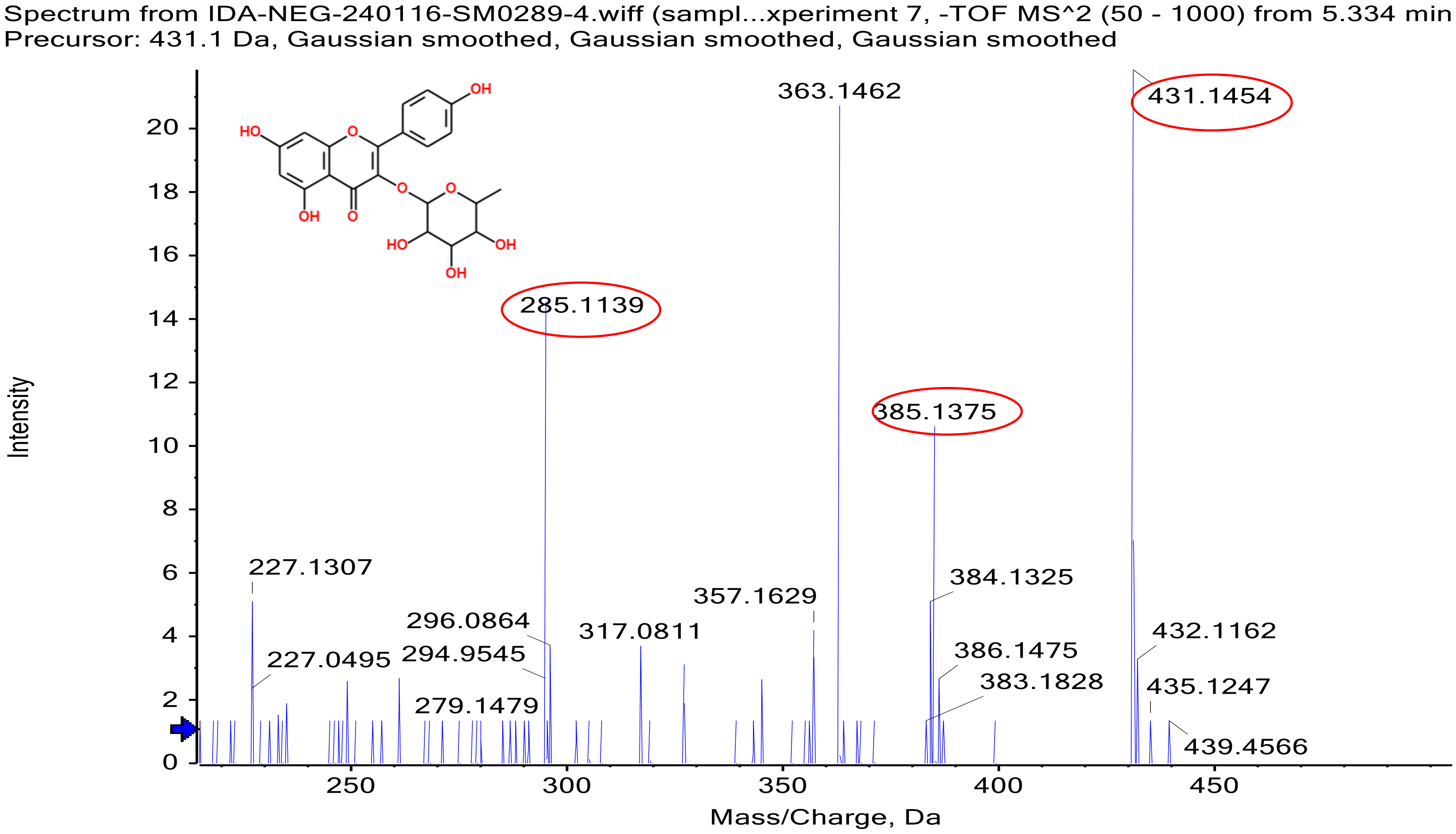

Figure (6S): LC/Ms-Ms of Kaempferol-*O*- deoxyhexoside of ETM in ESI negative mode

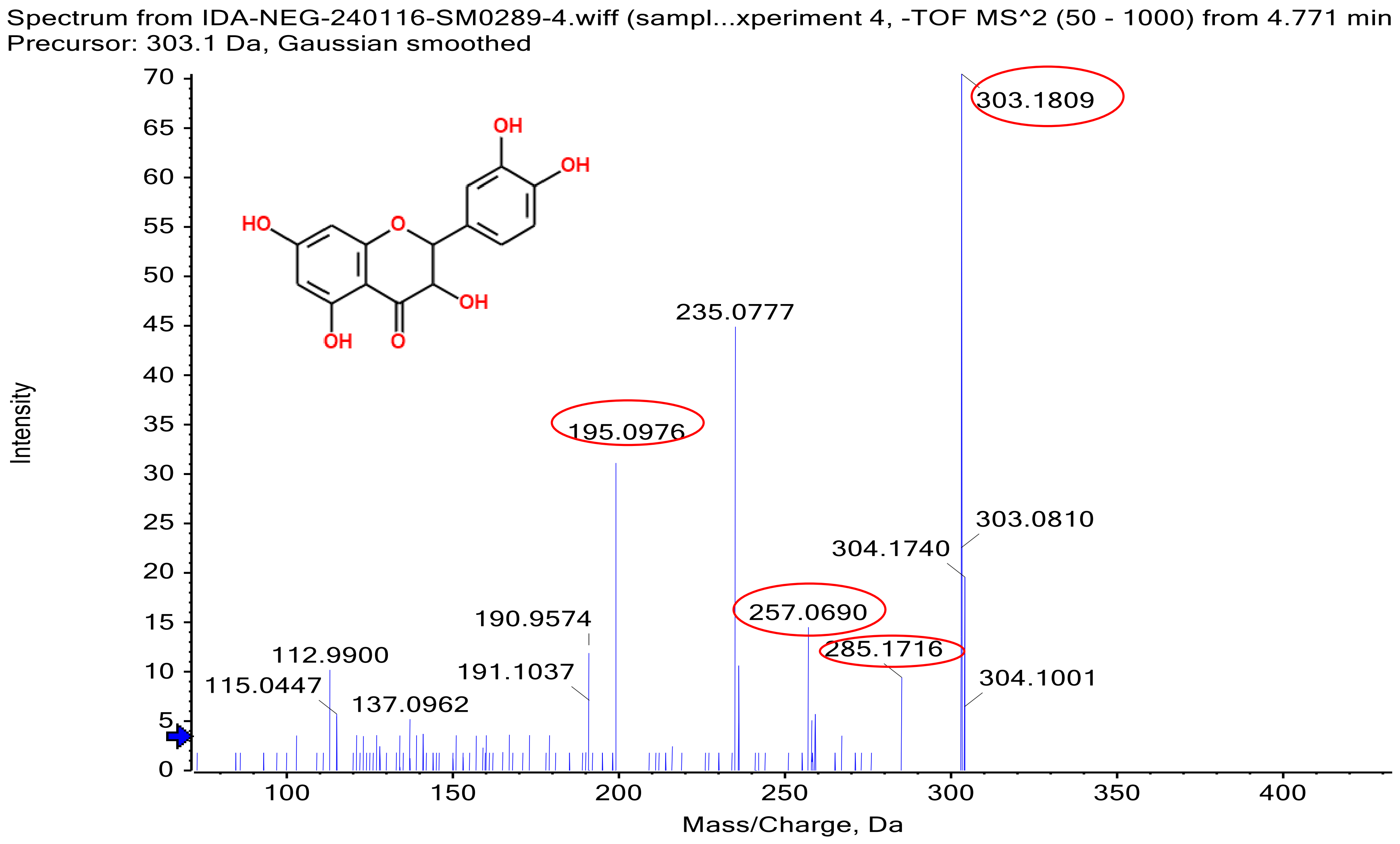

Figure (7S): LC/Ms-Ms of taxifolin of ETM in ESI negative mode

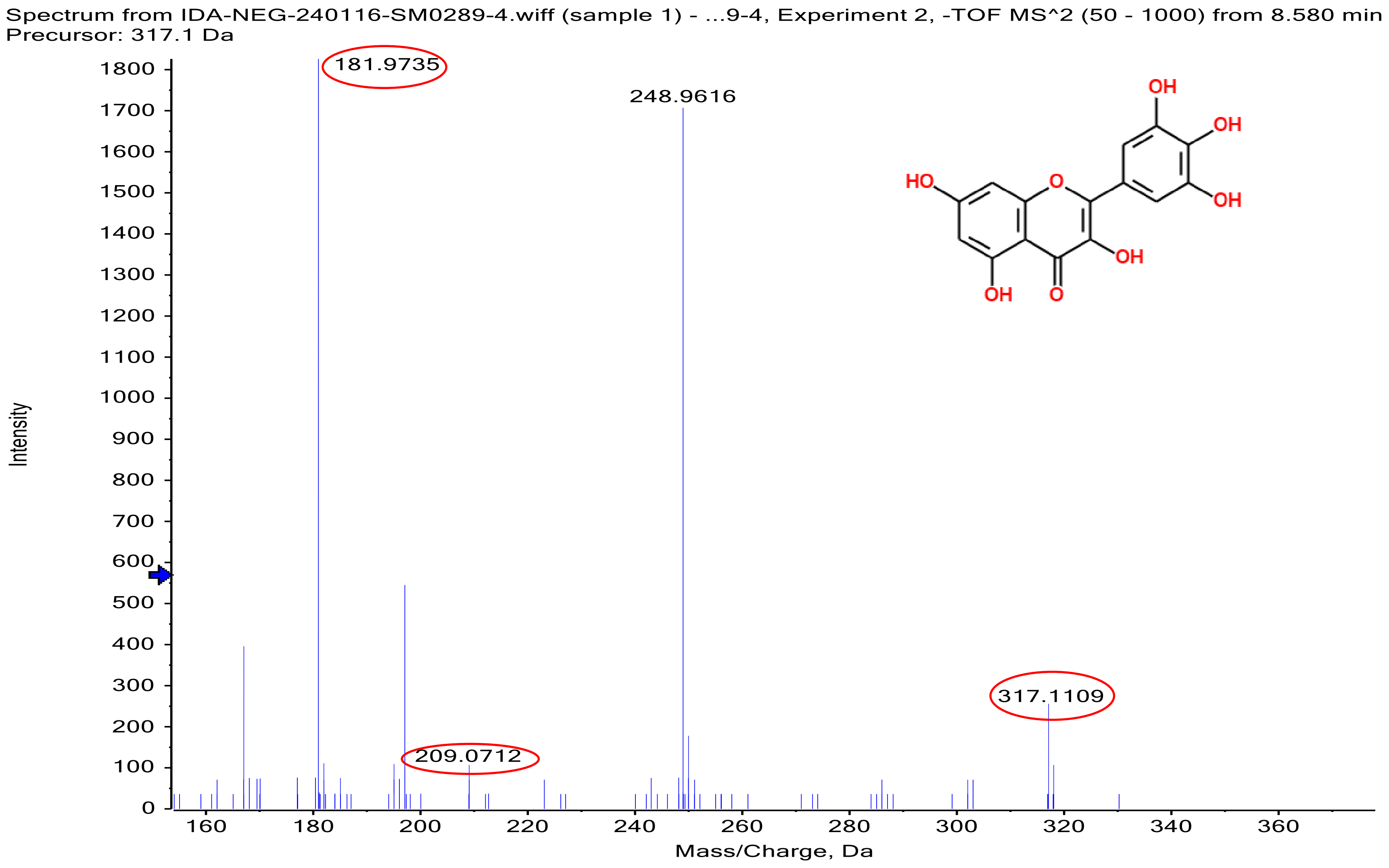

Figure (8S): LC/Ms-Ms of myrecetin of ETM in ESI negative mode

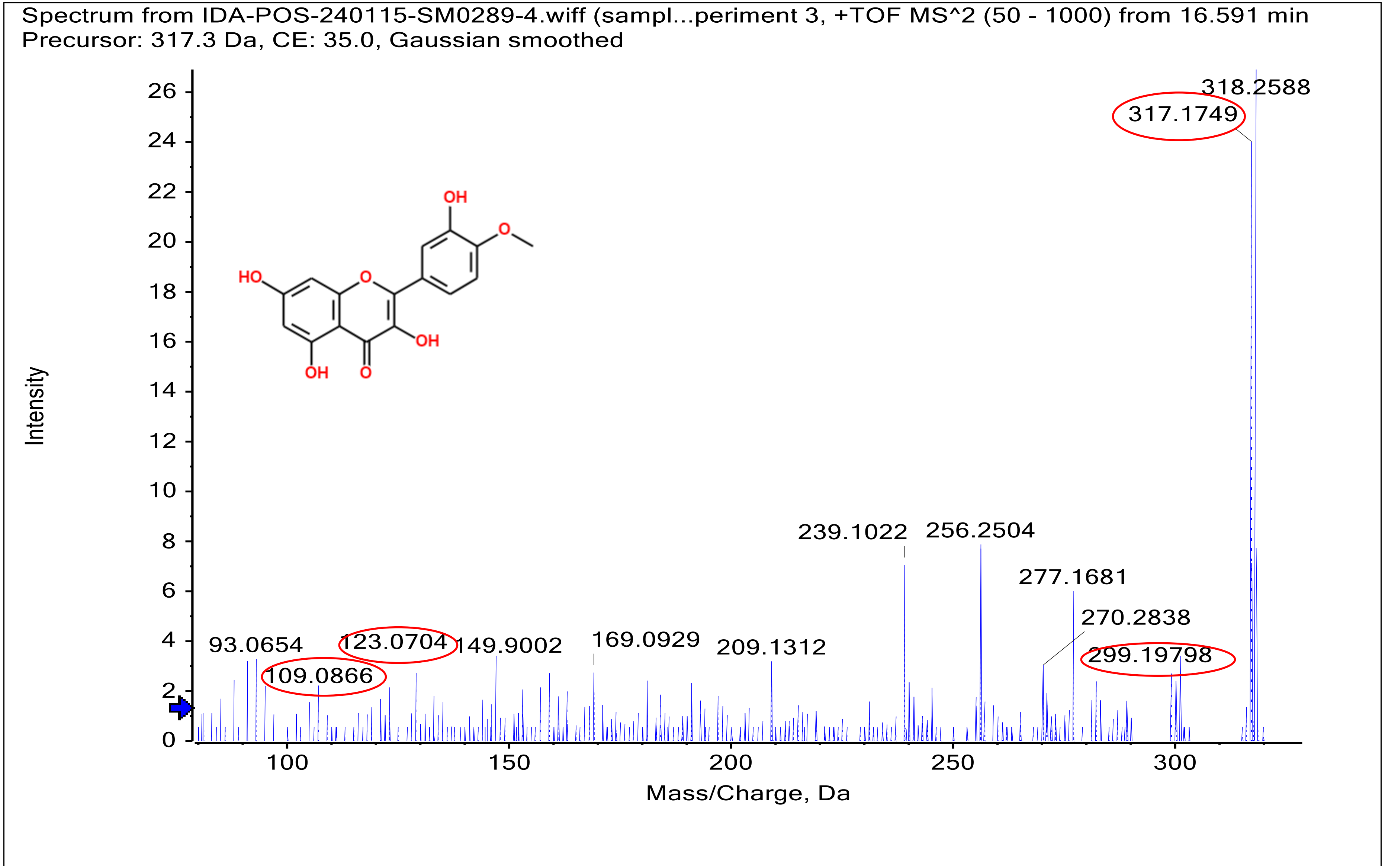

Figure (9S): LC/Ms-Ms of tetrahydroxy methoxy flavone of ETM in ESI positive mode

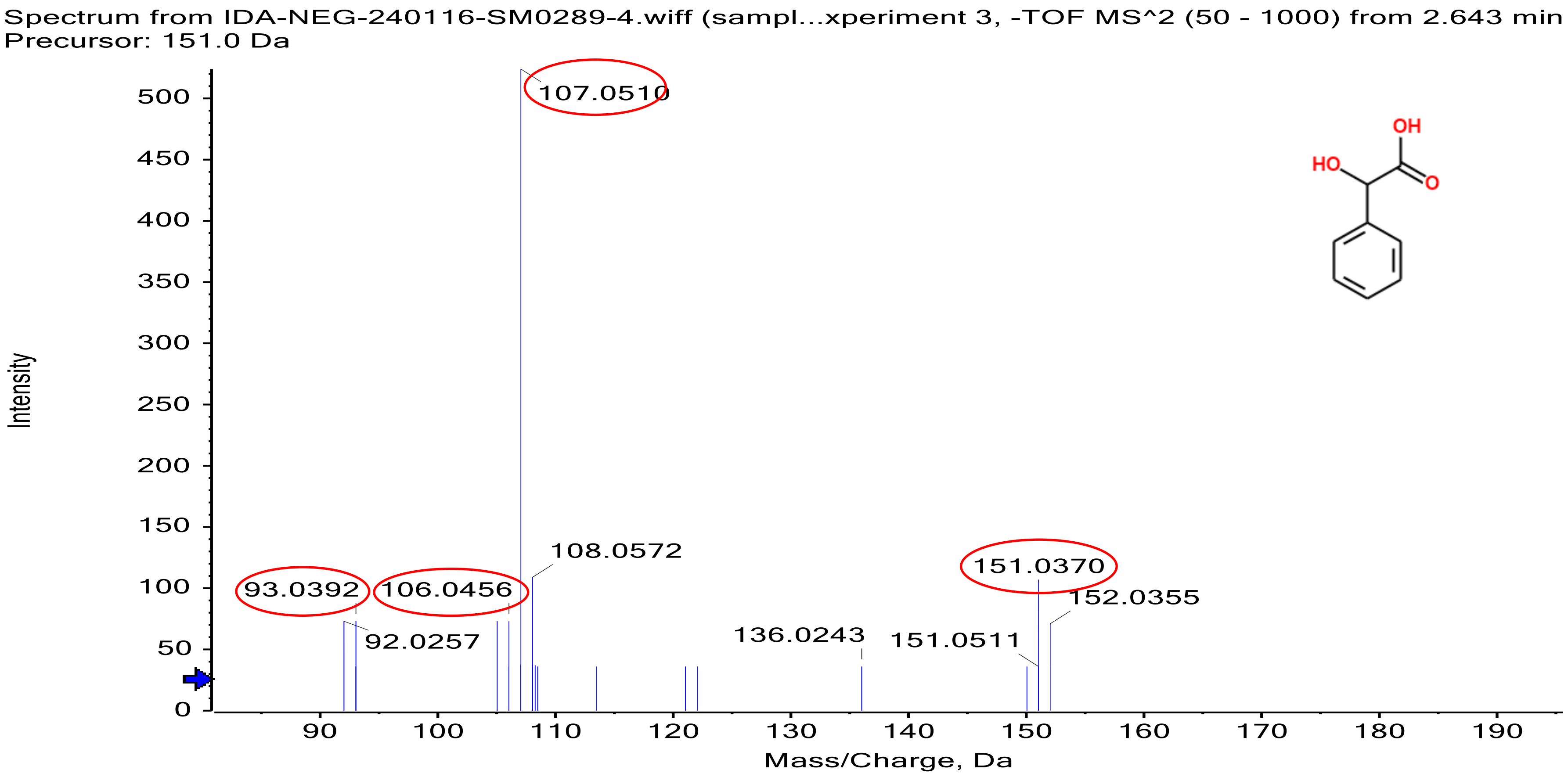

Figure (10S): LC/Ms-Ms of hydroxy phenylacetic acid of ETM in ESI negative mode

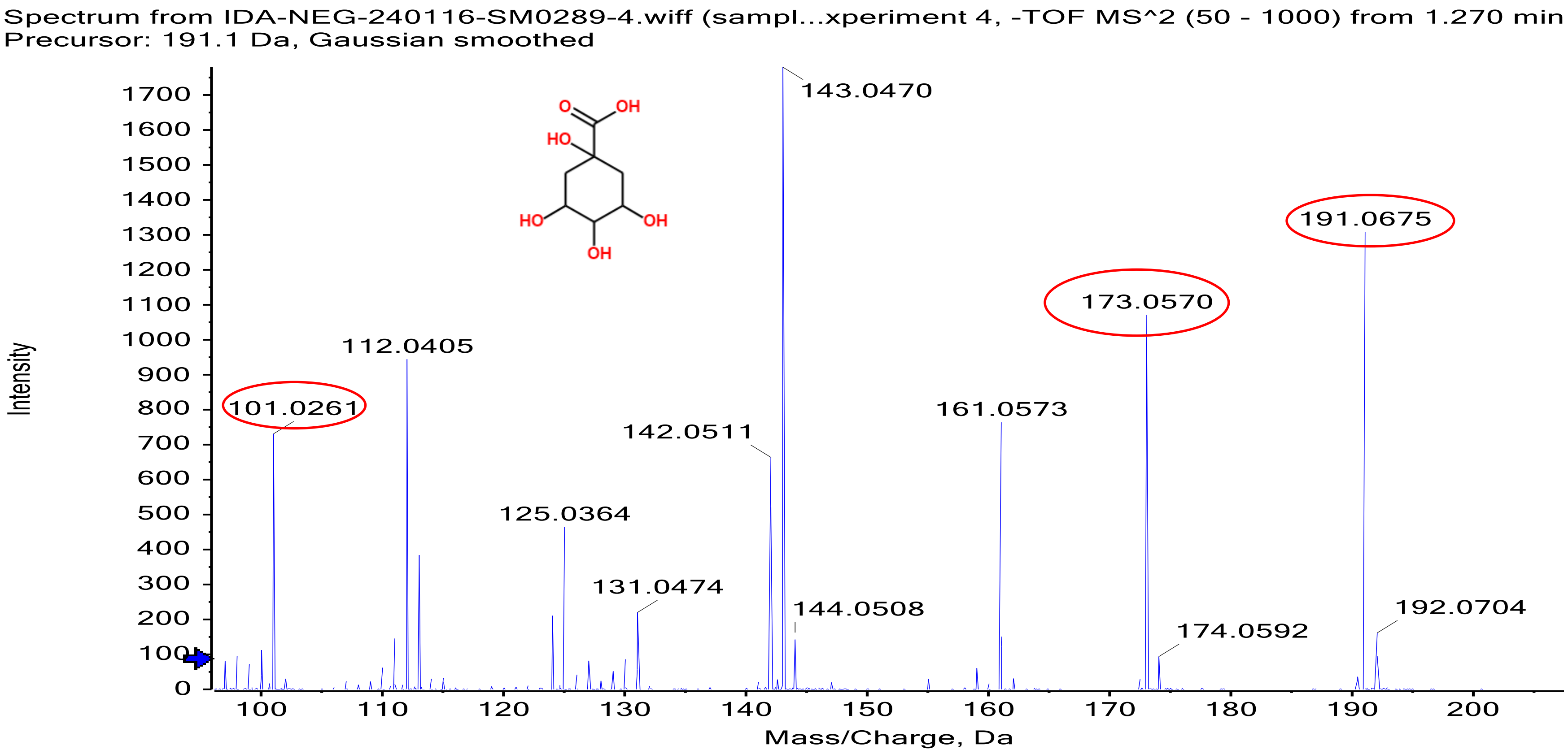

Figure (11S): LC/Ms-Ms of quinic acid of ETM in ESI negative mode

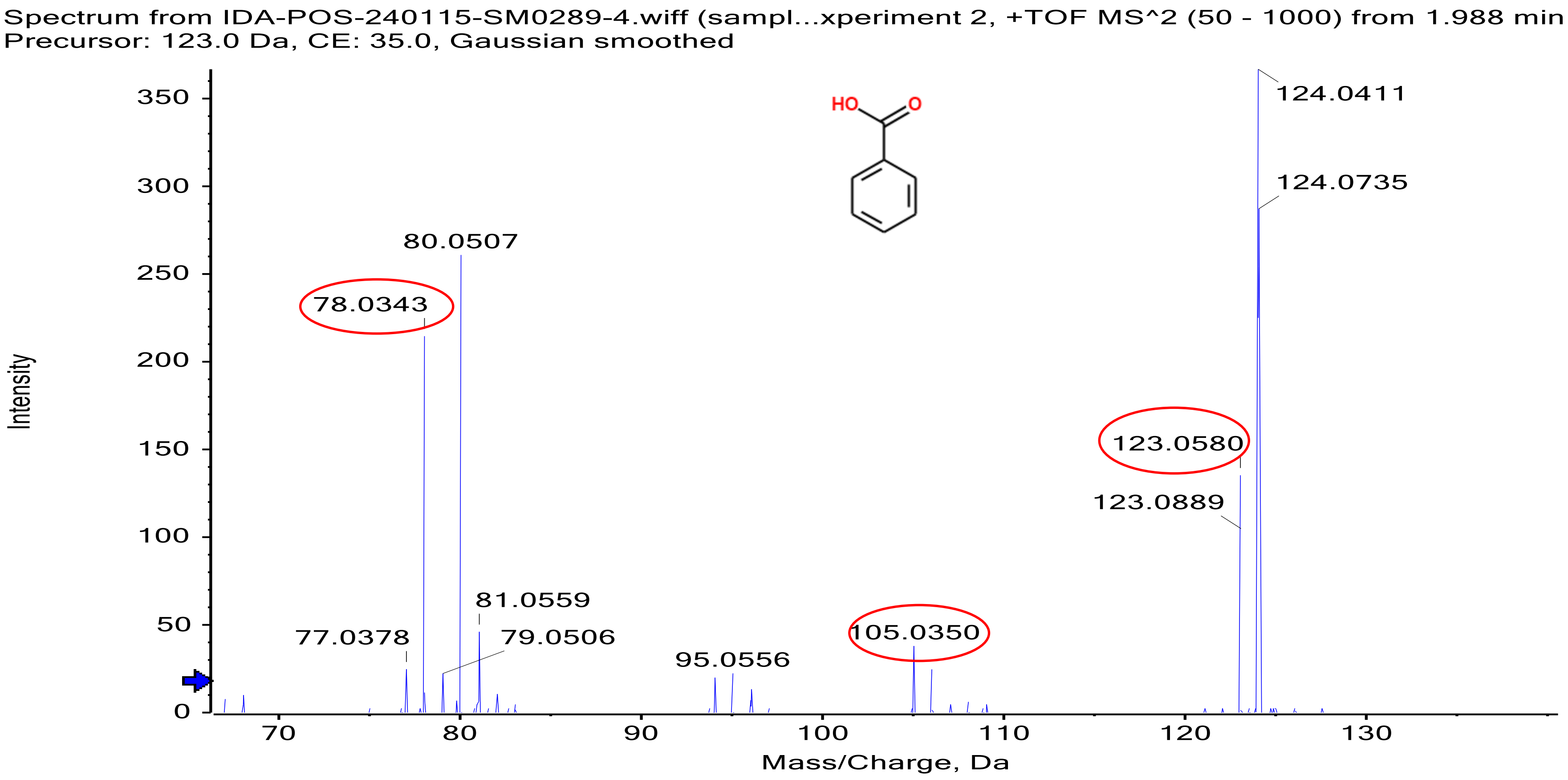

Figure (12S): LC/Ms-Ms of benzoic acid of ETM in ESI positive mode

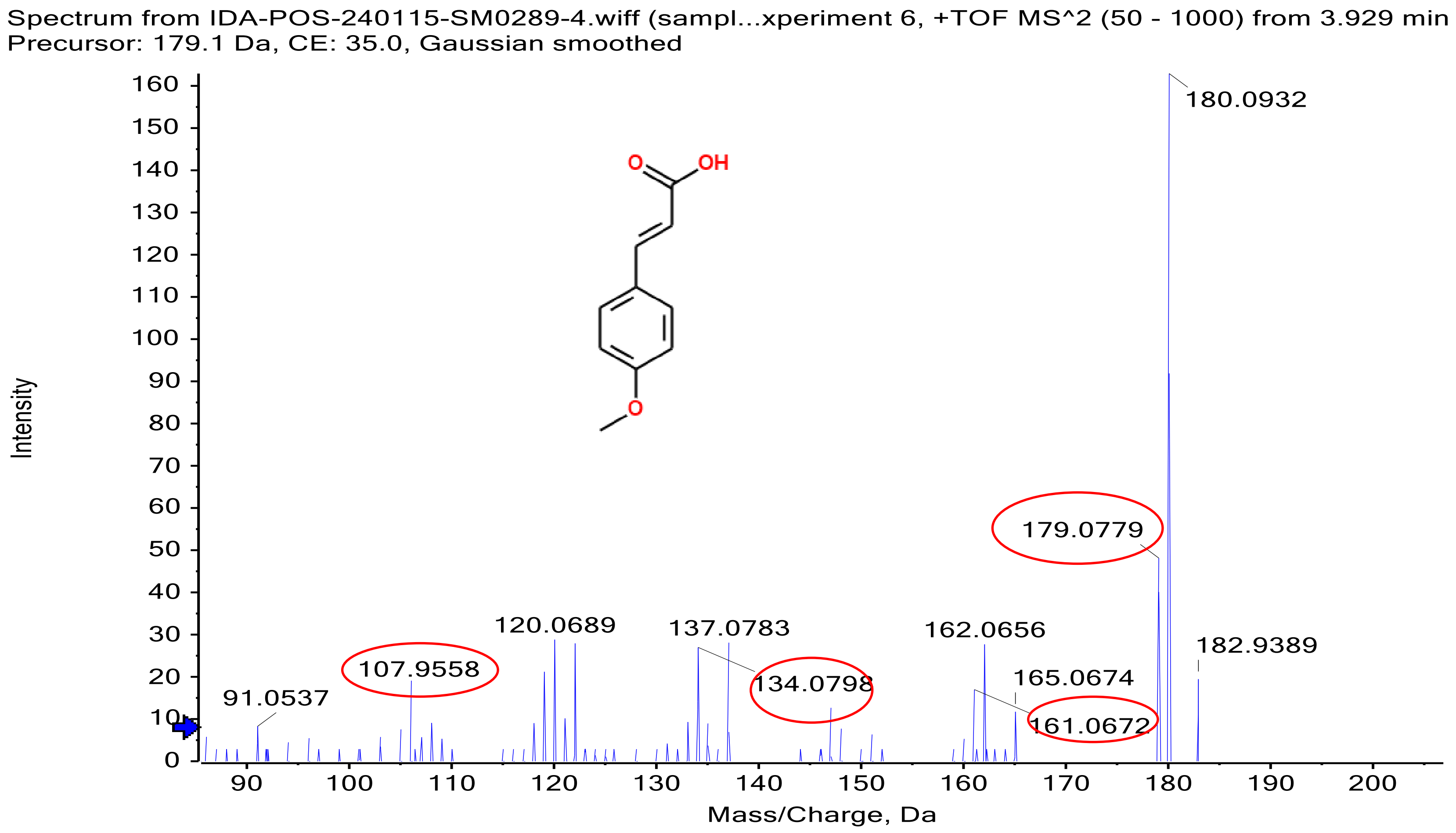

Figure (13S): LC/Ms-Ms of methoxy cinnamic acid of ETM in ESI positive mode

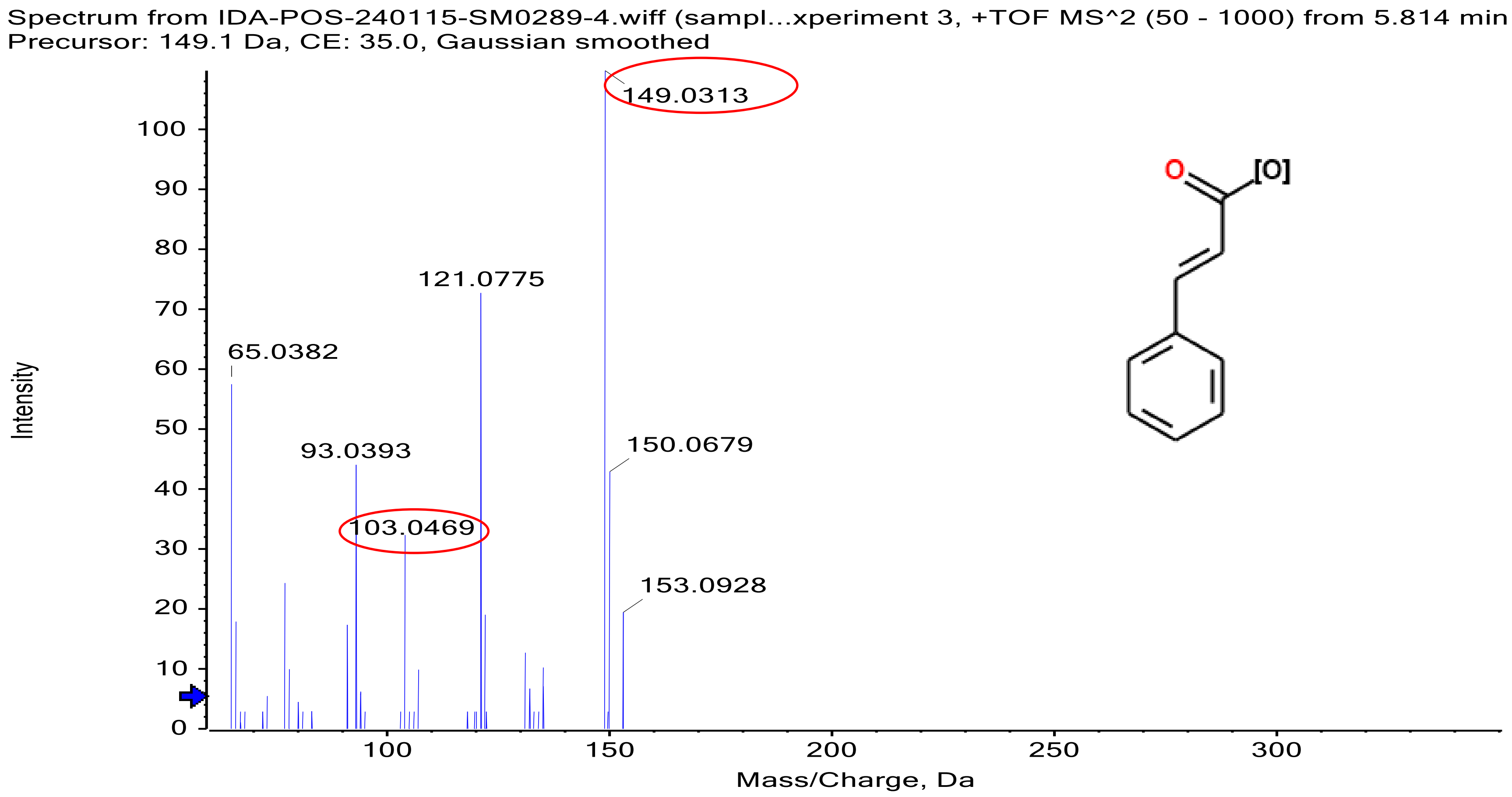

Figure (14S): LC/Ms-Ms of cinnamate of ETM in ESI positive mode
